# Knowledge, Attitude, and Behaviors Related to Eating Out among University Students in China

**DOI:** 10.3390/ijerph13070696

**Published:** 2016-07-12

**Authors:** Ping Hu, Wenjie Huang, Ruixue Bai, Fan Zhang, Manoj Sharma, Zumin Shi, Xiaoqiu Xiao, Abu S. Abdullah, Yong Zhao

**Affiliations:** 1 School of Public Health & Management, Chongqing Medical University, Chongqing 400016, China; m18323165365@163.com (P.H.); 15025357020@163.com (W.H.); bairuixue999@163.com (R.B.); ava11@126.com (F.Z.); 2 Research Center for Medicine and Social Development, Chongqing Medical University, Chongqing 400016, China; 3 The Innovation Center for Social Risk Governance in Health, Chongqing Medical University, Chongqing 400016, China; 4 Behavioral & Environmental Health, Jackson State University, Jackson, MS 39213, USA; manoj.sharma@jsums.edu; 5 Discipline of Medicine, University of Adelaide, Adelaide 5005, Australia; zumin.shi@adelaide.edu.au; 6 Laboratory of Lipid & Glucose Metabolism, The First Affiliated Hospital of Chongqing Medical University, Chongqing 40016, China; xiaoxq@cqmu.edu.cn; 7 Global Health Program, Duke Kunshan University, Kunshan 215347, China; Abu.Abdullah@bmc.org; 8 Duke Global Health Institute, Duke University, Durham, NC 27710, USA

**Keywords:** eating out, knowledge, attitude, behaviors, university students

## Abstract

In many countries the frequency of eating out has steadily increased over the last few decades, and this behavioris often associated with unhealthy dietary patterns. This study aimed to describe the levels of knowledge, attitude, and behaviors (KAB) related to eating out among university students. A cross-sectional study was conducted in the college town in Chongqing, China with a total of 1634 participants. The mean eating out related KAB scores were: knowledge 11.5 ± 2.9, attitude 17.0 ± 2.8, and behaviors 24.2 ± 4.8 (possible total scores: 20, 24, 40 respectively). As the level of knowledge increased, the percentage of highly satisfactory attitude and behaviors increased. Only 10% of the participants did not eat out for lunch and dinner during weekends in the last month. Gender, ethnicity, mother’s education, monthly boarding expenses, living place during the study, and the frequency of eating out for breakfast were statistically associated with the scores of KAB. In conclusion, Chinese junior students had poor knowledge of and behaviors towards eating out and ate out frequently. Educational interventionsto improve knowledge related eating out are needed in order to promote healthy eating out behaviors among Chinese university students.

## 1. Introduction

Obesity is a global public health concern that affects a third of the adolescent population in some countries [1]. Overweight and obesity have been associated with an increased risk of several chronic diseases, including type 2 diabetes, hypertension, cardiovascular diseases, and metabolic syndrome [2,3,4]. The main potential dietary determinants for overweight/obesity in adolescents are high intake of energy, low intake of dietary fiber and vegetables [5], and eating out of home [6].

In many countries, the frequency of eating out has steadily increased over the last few decades [7,8,9,10,11], and this increasing trend is expected to continue. Eating out is becoming particularly popular among young adults. A research study in the United States showed that young adults consumed approximately 40% of their total daily energy away from home [12]. In China, about 15% of the residents ate out of home every day [13]. Eating out is often associated with increased intake of energy [14,15,16] as well as fat and sugars [17,18]. This increased intake results in a low nutrient density [19], poor diet quality [15,20,21], and considerable weight gain [22,23,24,25].

Adolescent nutrition predicts the nutrition status of adults [26] and offers an important window of opportunity to prevent the risk factors for diet-related non-communicable diseases, which can be tracked later into adulthood [27]. When they enter universities, young adults become independent and develop their characteristics in a different social environment that often lead to different food choices and poor dietary habits [28].

To a certain extent, nutrition and food safety concerns are significant factors associated with the frequency of eating out among adolescents [29]. Eating out may encourage poor dietary patterns [30], as often it is associated with increased intake of energy. However, most people are unaware of the poor nutritional quality of foods consumed while eating out in unhealthy facilities or making unhealthy food choices. Understanding the knowledge, attitude, and behaviors (KAB) related to eating out could guide the development of appropriate educational intervention that would modify poor dietary patterns, and increase the awareness towards nutrition and food safety. Only a few studies have looked into KAB related to eating out among adolescents in China. Thus, our study aimed to determine the levels of KAB related to eating out, the frequency of eating out, and the factors associated with KAB among university students.

## 2. Materials and Methods

### 2.1. Study Design and Participants

In June 2014, a cross-sectional study was conducted in the college town in Chongqing, China. From 14 universities, two were randomly selected. At each selected university, two disciplines were then randomly selected. A total of thirty-six junior classes (freshmen and sophomores) in these disciplines were randomly considered. All students in the selected classes were invited to participate in the study. After omitting incomplete questionnaires, we included 1634 students in the analysis.

### 2.2. Definition of Eating Out

Based on the previous studies [8], “eating out” was defined as consumption of all foods not prepared at home or university campus canteen, irrespective of the place of consumption. If there are both home (canteen)-made food and non home (canteen)-made food, as long as the total energy mainly comes from non home (canteen)-made food, we also considered as “eating out” [31].

### 2.3. Survey Method and Outcome Measurements

#### 2.3.1. Survey Method

Taking class as a unit, the students were approached in their classroom before or after lectures. Students were asked to fill in a self-administered questionnaire, which took 15 to 20 min to finish. Afterwards a quick review of the questionnaire with the participants was performed by the investigators.

#### 2.3.2. Outcome Measurements-KAB Score

The self-administered questionnaire was developed based on the health KAB model [32] particularly designed for the target population. The final version of the questionnaire was obtained after a pilot study (25 students participated in the pretest) and repeated discussions with experts. The questionnaire had acceptable face and content validity. The internal consistency of the KAB questionnaire was acceptable (Cronbach’salpha = 0.80).

The knowledge of eating out was assessed by 20 single-choice questions covering issues of basic nutrition (e.g., healthy diet patterns, choosing the right type of foods, and avoiding foods with a high amounts of oils, salt, and fats)and food safety problems (e.g., expiry of food ingredients, bacteria). Each question was assigned a score of 1. The total score for this item could range from 0 to 20, a high score indicates a higher level of knowledge on the topic. Example of the questions included: “Do you think that there are a lot of bacteria on the menu”?

Eating out attitude was assessed by eight single-choice questions. Each question consisted of three levels with a score ranging from 1 to 3, which imply “without attention” to “attention”. The total score for this item may range from 8 to 24, and a high score indicates a good eating out attitude. Example of the asked questions for this item included: “How much attention did you pay to the dietary patterns while eating out”?

Eating out behavior was determined by 10 single-choice questions, such as “How often did you wash hands after touching the menu last month?” The participants were asked to provide a score (1 to 4) according to the frequency of these behaviors: 1 = never; 2 = occasionally; 3 = often; 4 = every time. The total score for this item may range from 10 to 40, and a high score indicates a good eating out behavior.

The total score of KAB was calculated based on the three above scores (ranging from 18 to 84). The scores of KAB were classified into five groups: “highly insufficient (≤20%)”, “insufficient (21%–40%)”, “general (41%–60%)”, “satisfactory (61%–80%)”, and “highly satisfactory (>80%)” [33,34].

### 2.4. Explanatory Variables

The socio-demographic information of the participants (i.e., gender, ethnicity, height, weight, body mass index (BMI), residence, parents’education, monthly living and boarding expenses, living place during the study and during holidays), and frequency of eating out were considered as the explanatory variables.

The participants were asked to report their respective height and weight. BMI was calculated as the ratio of weight (kg) to the square of height (m). The participants whose BMI < 18.5 kg/m^2^ were classified as thin, 18.5 ≤ BMI < 24 kg/m^2^ were regarded as normal, 24 kg/m^2^ ≤BMI < 28 kg/m^2^ were classified as overweight, and those with BMI ≥ 28 kg/m^2^ were considered as obese according to the Chinese criteria [35].

The participants were asked about their frequency as well as eating occasion (breakfast, lunch and dinner) of eating out during weekdays and weekends in the previous month.

### 2.5. Quality Assurance

All investigators for the study were recruited via interview to join the investigation team at the start of each term. The major teachers of these students gave them general training once or twice a month and specialized training prior to the implementation of each survey. Only investigators familiar with the approach, objectives, and methodology of the research as well as those who had experience in handling potentially sensitive issues were allowed to conduct the survey. 

### 2.6. Ethics Statement

This project was reviewed and approved by the Ethical Committee of the Chongqing Medical University (record number: 2013036) and was registered in the Chinese Clinical Trial Registry (Number: ChiCTR-OCH-14004861). A written informed consent was obtained from all participants. The participants were informed that they could withdraw from the study at any stage.

### 2.7. Statistical Analyses

Statistical analyses were performed using the SPSS V.17.0 software (SPSS Inc., Chicago, IL, USA). The descriptive data were expressed as mean ± standard deviation (SD) or proportions (%). Chi-square test was performed to test the differences among the categorical variables, and multiple linear regression analysis was used to assess the association between sociodemo graphic and lifestyle factors and KAB. The internal consistency of the questionnaire was calculated using Cronbach’s alpha. All statistics were analyzed through a two-sided test; a *p*-value that is less than or equal to 0.05 was considered statistically significant.

## 3. Results

### 3.1. Demographic Characteristics of the Study Sample

Table 1 shows the demographic characteristics of the study population. The majority (70.7%) of the respondents were female, three-fifths (59.4%) of them were from rural areas, and one-third (31.5%) had mothers with low education. The majority (71.1%) of the participants had monthly boarding expenses less than $100, and most of them (92.7%) lived in the dormitory during the study.

### 3.2. KAB Scores of Eating Out

**Figure 1:**
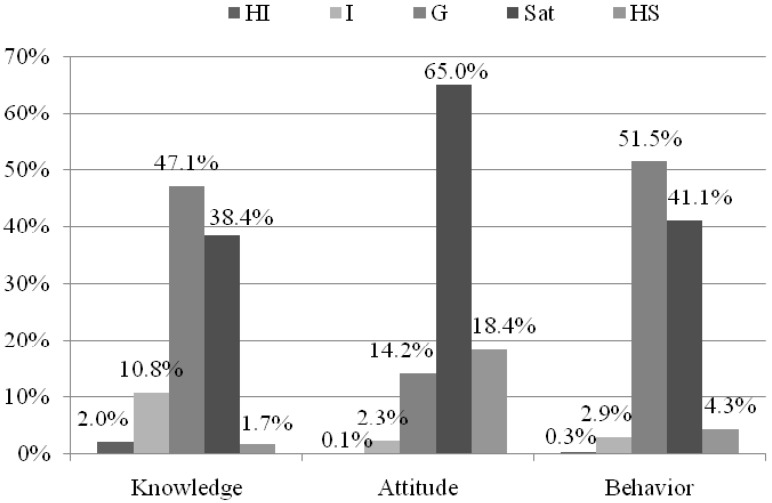
Distribution of eating out related KAB (*n* = 1634). HI, highly insufficient (≤20% of total score); I, insufficient (21%–40% of total score); G, general (41%–60% of total score); Sat, satisfactory (61%–80% of total score); HS, highly satisfactory (>80% of total score).For knowledge: N_HI_ = 33; N_I_ = 176; N_G_ = 769; N_Sat_ = 628; N_HS_ = 28. For attitude: N_HI_ = 2; N_I_ = 38; N_G_ = 232; N_Sat_ = 062; N_HS_ = 300. For behavior: N_HI_ = 5; N_I_ = 47; N_G_ = 841; N_Sat_ = 671; N_HS_ = 70.

The mean score of eating out related knowledge, attitude and behaviors was 11.5 ± 2.9, 17.0 ± 2.8, and 24.2 ± 4.8, respectively.The levels of KAB were classified into the five categories (Figure 1): HI (highly insufficient), I (insufficient), G (general), Sat (satisfactory), and HS (highly satisfactory). The quintile (first to fourth) scores for knowledge were 4, 8, 12, and 16; for attitude were 5, 10, 14, and 19; and for behaviors were 8, 16, 24, and 32. The distribution of eating out related knowledge were: highly insufficient (2.0%), insufficient (10.8%), general (47.1%), satisfactory (38.4%), and highly satisfactory (1.7%). The percentage of students with satisfactory and highly satisfactory attitude were 65.0% and 18.4%, respectively, while less than 3% had insufficient or highly insufficient attitude. Less than half of the students had highly satisfactory (4.3%)/satisfactory (41.1%) behaviors.

Among participants with satisfactory knowledge, 65.9% had satisfactory attitude and 45.9% had satisfactory behaviors (Table 2). Among participants with highly satisfactory knowledge, 28.6% had highly satisfactory attitude and 10.7% had highly satisfactory behaviors. In contrast, among those with highly insufficient knowledge, only 6.1% had highly satisfactory attitude and highly satisfactory behaviors.The highly satisfactory attitude of the participants increased from 6.1% to 28.6%, and their highly satisfactory behaviors increased from 6.1% to 10.7% with the increase level of knowledge.

### 3.3. Frequency of Eating Out

Table 3 shows that 22.9% of the participants ate out for breakfast more than three times per week during the previous month. Males were more likely to eat out than females (*p* = 0.013). For lunch and dinner, a third of the participants ate out more than three times per week during weekdays. Only 10% of the participants did not eat out for lunch and dinner during weekends.

### 3.4. Factors Associated with KAB of Eating Out

In the univariate analyses, gender, ethnicity; residence; parents’ education; monthly boarding expenses; living place during the study and frequency of eating out for breakfast were significantly associated with KAB of eating out (data not shown). All these variables were included in a multiple regression model to identify the predictors of KAB (Table 4).

Table 4 shows that females had a higher scores than males (β *=* 0.103, *p* < 0.001). However, no gender difference of attitude and behaviors scores was observed. BMI, residence and father’s education were not associated with any components of KAB. Monthly boarding expense was positively associated with knowledge score (β = 0.053, *p* = 0.044), while mother’s education was positively associated with behaviors score (β *=* 0.106, *p* ≤ 0.001). Minority participants had a lower knowledge score than Han participants (β *=* −0.054, *p* = 0.032). Participants who lived in school during the study had a higher knowledge score than other participants (β *=* −0.115, *p* < 0.001). Frequency of eating out for breakfast was inversely associated with knowledge (β *=* −0.113, *p* < 0.001) and attitude (β *=* −0.100, *p* ≤ 0.001) scores, while it was positively associated with behaviors scores (β *=* 0.071, *p* = 0.006). 

## 4. Discussion

In this cross-sectional study, we found that Chinese junior students had poor knowledge of and behaviors towards eating out and that they frequently ate out.This study was the first of its kind to assess KAB related to eating out among university students in Chongqing, China. The findings provide entry points for promoting healthy eating among university students in China.

Extensive increase in KAB levels related to eating out can help modify one’s poor dietary patterns, and increase one’s awareness toward nutrition and food safety of eating out in unhealthy premises or making unhealthy food choices. Our study revealed that university students had a low levels of knowledge and behaviors related to eating out, despite the fact they had a relatively good attitude. Earlier studies in China showed that eating out related KAB among those who frequently eat out was poor [36]. Some studies also discovered that adolescents had low health concerns while choosing foods [37,38]. Direct comparison with other studies is difficult due to the differences of demographic characteristics of the study sample as well as the measurements used. Most junior university students often have good attitude towards positive things and have strong desire for improved knowledge.

Eating out significantly affects the food choices of adolescents [39]. A good command of nutritional knowledge is important for choosing the healthy food and maintaining a healthy weight as well as general health [40]. In our study, only a few participants had satisfactory knowledge and behaviors towards eating out. This phenomenon may be attributed to several reasons: Firstly, most junior students have left home and are living independently for the first time. Secondly, they may experience great peer pressure for eating out. Only a few had taken a course on nutrition and food safety related to eating out. One study has shown that systematic and proper nutrition education can help increase the KAB level of those who frequently eat out [36]. Our study suggests a substantial need for eating out-related intervention among university students.

A lack of nutrition and food safety concerns has been shown to be one of the significant factors associated with the frequency of eating out [29]. Our result showed that the frequency of eating out of home among Chinese university studentswas high. The prevalence of eating out of home in this study is higher than that in the results of the 2002 China National Nutrition and Health Survey [41] and the 2011 China Health and Nutrition Study [42]. For breakfast, the frequency of males eating out was higher than that of females, whereas the frequencies of eating out for lunch and dinner had no statistical significance. This observation is partially consistent with previously studies which suggested that men tend to consume more of their total dietary energy away from home than women [14,43]. Different from our study, one study in UK found that women ate out as frequent as men [44]. 

In our study, females tend to display better nutrition and food safety knowledge than males. This result is consistent with the findings of other studies [40,45]. This gender difference may due to the fact that girls pay more attention to the quality of food, whereas boys eat out with their peers more frequently for dinner. Girls also care more about body image than boys.

Eating is a social event; thus, the presence of family and friends can inevitably affect food choices [46]. Numerous epidemiological studies have shown that parental education had a positive independent effect on the quality of children’s diet [47,48]. Our data also indicated that students attained a high score on behaviors when their mother’s education level is high. By contrast, no significant association between the father’s educational level and the KAB scores was observed. These observations imply that mothers significantly influence adolescents’ nutrition because they remain as the core person in the family who chooses and prepares the food. High parental education level may increase the children’s motivation to follow a healthy lifestyle.

We found an inverse association between the frequency of eating outfor breakfast and the scores of knowledge and attitude. This findingsuggests that future intervention should target those who frequently ate out.

The university students’ KAB levels related to eating out varied. In particular, the students’attitude and behaviors increased with the increase in knowledge, and their behaviors increased with the increased level of attitude towards eating out. The KAB model consider that knowledge is essential for effective changes in behaviors, attitude is the driving forces for modifying behaviors, and that students can obtain knowledge and skills through learning. Our study implies that their knowledge and behaviors levels towards such practice are poor and that Chinese university students frequently eat out. Therefore, information interventions about eating out should improve the knowledge of university students and guide their related behaviors.

### Limitations

Our study has several limitations that should be considered. First, only a few validated and reliable questionnaires are available in the field of eating out and making healthy food choices. The questionnaire adopted for this study was self-designed by our team after a pretest and repeated discussions with experts. Second, we do not have information on the actual food intake in the study, we only assessed the baseline eating out KAB of university students to aid the planning of health promotion activities. Third, we acknowledge the fact that eating out itself is not a major problem, but going to unhealthy food premises or making poor food choices are problems. Understanding of both components would have provided detailed information to support the development of intervention.

## 5. Conclusions

Our study demonstrated that knowledge and behaviors related to eating out among university students in China were poor and the frequency of eating out among this population was relatively high. Educational interventions for eating out should improve the knowledge of university students and guide their related behaviors.

## Figures and Tables

**Table 1:** Demographic characteristics of the study population (*n* = 1634).

Demographic Variables		Total (*n* = 1634)	
		*n*	%
Gender	Male	478	29.3
	Female	1156	70.7
Ethnicity	Han	1430	87.5
	Minority	204	12.5
BMI	Thin (<18.5 kg/m^2^)	338	21.1
	Normal (18.5–23.9 kg/m^2^)	1169	72.8
	Overweight (≥24 kg/m^2^)	87	5.4
	Obesity (≥28 kg/m^2^)	11	0.7
Residence	Urban	642	40.6
	Rural	940	59.4
Father’s education	Primary school or below	300	18.5
	Middle school	688	42.4
	High school/secondary	454	28.0
	College education or more	181	11.1
Mother’s education	Primary school or below	512	31.5
	Middle school	639	39.4
	High school/secondary	363	22.4
	College education or more	110	6.7
Monthly boarding expenses	<$100	1153	71.1
	$100–$150	405	25.0
	≥$150	64	3.9
Living place during the study	Dormitory	1512	92.7
	Not in dormitory	119	7.3

Data were expressed as *n* (%).

**Table 2:** Percentage distribution of eating out related KAB (*n* = 1634).

Knowledge (%)	Attitude (%)				
	HI	I	G	Sat	HS
HI	6.1	24.2	27.3	36.4	6.1
I	0	5.1	27.8	58.0	9.1
G	0	2.0	14.3	67.4	16.4
Sat	0	1.0	9.6	65.9	23.6
HS	0	0	14.3	57.1	28.6
**Knowledge** **(%)**	**Behavior** **(%)**				
	**HI**	**I**	**G**	**Sat**	**HS**
HI	6.1	9.1	45.5	33.3	6.1
I	0.6	6.3	50.6	38.6	4.0
G	0.3	2.6	55.0	37.8	4.3
Sat	0	1.9	48.2	45.9	4.0
HS	0	3.6	39.3	46.4	10.7
**Attitude** **(%)**	**Behavior** **(%)**				
	**HI**	**I**	**G**	**Sat**	**HS**
HI	100	0	0	0	0
I	0	13.2	57.9	15.8	13.2
G	0	7.3	61.2	29.3	2.2
Sat	0.3	2.0	54.5	40.6	2.6
HS	0	1.3	32.7	55.3	10.7

HI, highly insufficient; I, insufficient; G, general; Sat, satisfactory; HS, highly satisfactory.

**Table 3:** Frequency of eating out per week in the last month by gender (*n* = 1634).

Frequency of Eating Out		Total	Male	Female	*p* ^1^
		*n* (%)	*n* (%)	*n* (%)	
Breakfast (Mondays to Sundays)	0	661 (40.6)	168 (35.2)	493 (42.8)	0.013 *
	1–3	596 (36.6)	196 (41.1)	400 (34.7)	
	>3	373 (22.9)	113 (23.7)	260 (22.5)	
Weekdays (Lunch and Dinner)	0	279 (17.1)	80 (16.7)	199 (17.3)	0.950
	1–3	813 (49.9)	238 (49.8)	575 (49.9)	
	>3	538 (33.0)	160 (33.5)	378 (32.8)	
Weekends (Lunch and Dinner)	0	158 (9.8)	55 (11.7)	103 (9.0)	0.252
	1–2	996 (61.9)	285 (60.8)	711 (62.3)	
	3–4	456 (28.4)	129 (27.5)	327 (28.7)	

^1^ Obtained from a Chi-square test. * *p* < 0.05 (significant difference).

**Table 4:** Multiple linear regression analysis with background and KAB.

Parameter	Knowledge			Attitude			Behavior		
	β	SE	*p*	β	SE	*p*	β	SE	*p*
Gender (Male(0), Female(1))	0.103	0.163	<0.001 *	0.054	0.160	0.041	0.004	0.271	0.886
Ethnicity (Han(0), Minority(1))	−0.054	0.217	0.032 *	0.008	0.214	0.762	−0.027	0.362	0.282
BMI	0.016	0.033	0.541	0.001	0.032	0.976	0.043	0.055	0.099
Residence (Urban(0), Rural(1))	−0.056	0.170	0.056	0.002	0.167	0.959	0.032	0.284	0.289
Father’s education (Low education(0), Higheducation(1))	0.055	0.184	0.079	0.027	0.181	0.404	0.013	0.306	0.677
Mother’s education (Low education(0), High education(1))	0.031	0.198	0.330	0.004	0.195	0.900	0.106	0.331	0.001 *
Monthly boarding expenses (<$100(0), $100–$150(1), ≥$150(2))	0.053	0.136	0.044 *	−0.043	0.134	0.108	−0.020	0.227	0.447
Living place during the study (Dormitory(0), Others(1))	−0.115	0.275	<0.001 *	−0.038	0.271	0.141	0.038	0.459	0.138
Frequency of eating out for breakfast (≤3(0), >3(1))	−0.113	0.172	<0.001 *	−0.100	0.169	<0.001 *	0.071	0.287	0.006 *

β indicates standardized partial regression coefficient; SE, indicates standard error; BMI, body mass index. * *p* < 0.05 (significant difference).

## Abbreviations

The following abbreviations are used in this manuscript:

KAB	knowledge, attitude, and behaviors
HI	highly insufficient
I	insufficient
G	general
Sat	satisfactory
HS	highly satisfactory
